# Imported COVID-19 Cases from Iran: A Rapid Review

**DOI:** 10.3390/epidemiologia2020015

**Published:** 2021-05-29

**Authors:** Tasnim Abdalla, Khaled Aboujabal, Menatella Abdelnaby, Rim Bashir, Yara Wanas, Elmoubasher Farag

**Affiliations:** 1 College of Medicine, Qatar University, Doha, Qatar; ka1605056@student.qu.edu.qa (K.A.); ma1602471@student.qu.edu.qa (M.A.); rb1601555@student.qu.edu.qa (R.B.); yw1508597@student.qu.edu.qa (Y.W.); 2 Department of Public Health, Ministry of Public Health, Doha, Qatar; eabdfarag@moph.gov

**Keywords:** COVID-19, Iran, travel, imported cases, quarantine, epidemiology

## Abstract

This review aims to map the spread of the virus from Iran to the Middle East and the rest of the world and to help better understand the key trends that occurred during COVID-19 from this epidemic center. We performed a literature review which was undertaken from 16 June to 22 November 2020. We reviewed the available evidence on imported cases from Iran, in the electronic databases PubMed and Google Scholar, as well as gray literature. It is shown that 125 cases were imported from Iran, out of which most of the imported cases were asymptomatic, and PCR testing was the most common method of detection. It was also found that more than half of the imported cases were not quarantined or isolated at home. The review revealed that many countries, especially the Middle East had imported cases from Iran. The big gap between the date of arrival at the airport and the date of diagnosis emphasizes the importance of early detection and quarantine measures, to stop the spread of the virus.

## 1. Introduction

During the early stages of COVID-19 pandemic in March 2020, there were three major epidemic centers: China, Italy, and Iran. The first two reported cases in Iran were imported from China. Shortly afterward all the provinces had been affected, and the rates ranged from 0.8 to 61.8 cases per 100,000 population. The highest number of cases have been reported in Iran’s capital and most populated city of Tehran (1945), followed by the city of Qom (712), which has a significant religious site for mass gatherings, and thirdly in Mazandaran (633), a popular destination for spring tourism [1]. 

Several key factors contributed to a rapid increase in the number of cases in Iran. The early stages of the outbreak coincided with national events including the anniversary of the Iranian revolution (11 February 2020), the eleventh parliament election (21 February 2020), as well as the Nowruz celebration (a 2-week celebration of the Iranian new year that started on 20 March 2020). Furthermore, due to the limited understanding of the magnitude of the infection and its impact, there were delays in the implementation of preventative measures, such as closure of religious sites, suspension of schools, and disinfection of public transportation [1]. Moreover, some religious sites were exempted from closures. In addition, there were apparent delays in reporting of official data and information from within the country. Lastly, sanctions imposed on Iran from the international community caused a shortage of medical supplies, and Iran has an economic and strategic partnership with China where travel and trade can increase their risk for imported cases [2].

As of 17 June, Iran reported around 200,000 confirmed cases of the infection and 9000 deaths [3]. The first reported case was on the 20 February, then the first peak was on 5 April, with approximately 33,000 active cases. Thereafter, there was a steady decline in cases until 3 May, where it reached almost 13,000 active cases, and a steady incline was observed, reaching the 2nd peak on the 20 June with around 32,000 active cases. Even though the number of active cases decreased for a few months after that, the number of active cases in Iran is steadily increasing, with a peak on 14 April 2021, recording 25,582 active cases [4].

Accordingly, Iran became the main source of spread of disease to the Middle East through imported cases [4]. Previous studies have aimed to describe the problem of imported diseases even before the emergence of the COVID-19 pandemic; Napoli C. et al described the imported cases of Chikungunya (CHIKV) and Dengue (DENV) virus in Italy between 2008–2011 and found that 130 cases of CHIKV and DENV have been imported from Mauritius, Maldives, Bali, Sri Lanka, and Asia during that period [5].

In a similar fashion, we are aiming to describe the imported cases of COVID-19 in the middle east region. For the Middle East region, the epidemic center through which cases were imported from is Iran, and this is important to monitor as many neighboring countries have family and economic ties with Iran. Moreover, there seems to be a lack in the studies that focused on tracing the cases imported from Iran. This study aimed to review reports of imported cases from Iran and to map the spread of the virus to the Middle East and the rest of the world. A Better understanding of key trends and patterns that occurred during COVID-19 from this epidemic center will help inform neighboring countries in ongoing and future emergency preparedness for communicable diseases. It will also help in taking the necessary public health measures in order to curb the spread of the virus, such as travel restrictions, airport screening, and quarantining protocols.

## 2. Materials and Methods

A systematic literature search was conducted in PubMed and Google Scholar in June 2020. The following search terms were included: “imported” “SARS-CoV-2” from “Iran” in both electronic databases. The authors also scanned the reference lists of the included studies and the relevant review papers, which included both journal articles and “Gray Literature”, which were articles from news and media outlets, as well as World Health Organization (WHO) reports, that shared the numbers of imported cases, and other information related to the cases. However, we limited the use of Gray Literature to only in situations where we needed to acquire data for countries that did not have articles in the literature regarding imported cases from Iran. During the literature search, titles and abstracts were screened from various articles and full-text reports were also reviewed and selected in accordance with the following inclusion criteria: (1) Studies that reported cases of COVID-19 imported from Iran; (2) Both individual and group cases of COVID-19; (3) Cases were infected by COVID-19 in Iran only and then imported somewhere else; (4) Studies that included patient-level data. Exclusion criteria: (1) Articles that did not contain appropriate and sufficient data regarding the imported cases of COVID-19 from Iran; (2) Studies not written in English.

**Figure 1:**
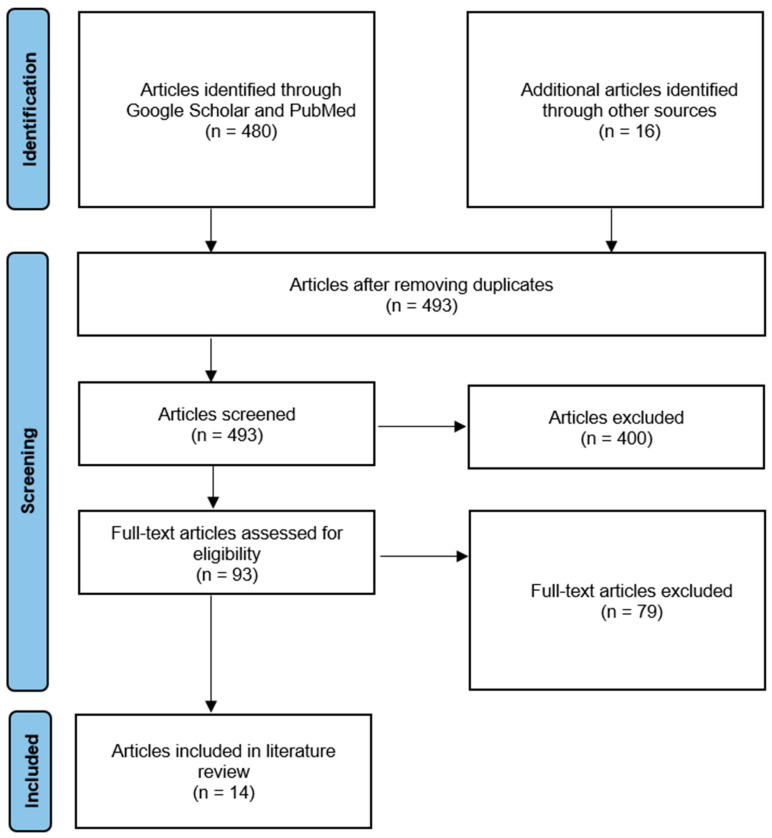
The figure shows a flowchart of the inclusion and exclusion criteria of the articles.

Data extracted included the age, sex, nationality, symptoms, risk factors, date of arrival, date of symptoms’ onset, date of diagnosis, detection location, detection mechanism, if they were in home isolation or quarantine, and whether they had caused any secondary infection. We summarized the following (1) the total number of cases found by the review imported from Iran, (2) the total number of countries, (3) the dates when cases were imported into other countries, and (4) available data on the characteristics of these cases. The database search shown in Figure 1 identified a total of 496 possible relevant articles. Following the removal of duplicates, 493 were included for further screening. Under inclusion and exclusion criteria mentioned previously, 93 were qualified after excluding 400 articles. Further 79 articles were eliminated after reviewing full text articles based on inclusion and exclusion criteria, and a total of 14 articles were included in the literature review.

This process was done by fairly splitting the number of articles for each of the authors. Then an online document was created with a list of each of the articles that each must screen, and the authors would write the qualified articles’ title, authors and DOI in another online document. The 493 articles on the online document were then re-examined to ensure that they had all been screened.

## 3. Results

### 3.1. Countries with Imported Cases

**Figure 2:**
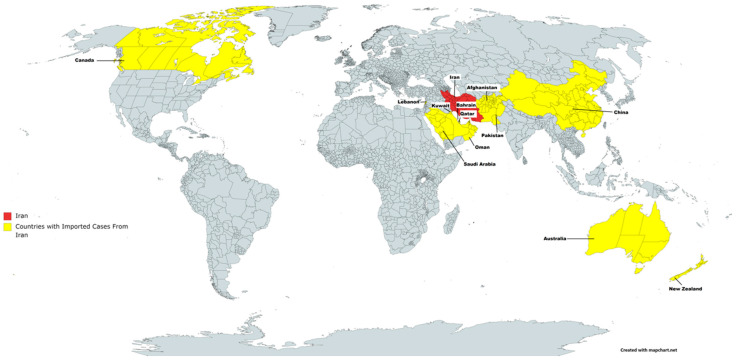
Map showing countries that had imported COVID-19 cases from Iran. The Islamic Republic of Iran is highlighted in red and countries with reported COVID-19 cases from Iran in yellow.

A map showing the countries or cities that had imported cases from Iran is shown in Figure 2. There was a total of 70 cases imported from Iran around the world in 14 countries which can be seen in Table 1: United Arab Emirates (11), Bahrain (26), Oman (3), Kuwait (5), Saudi Arabia (5), China (4), Canada (3), Lebanon (3), Qatar (3), Pakistan (2), United States of America (2), Afghanistan (1), Australia (1) and New Zealand (1).

**Figure 3:**
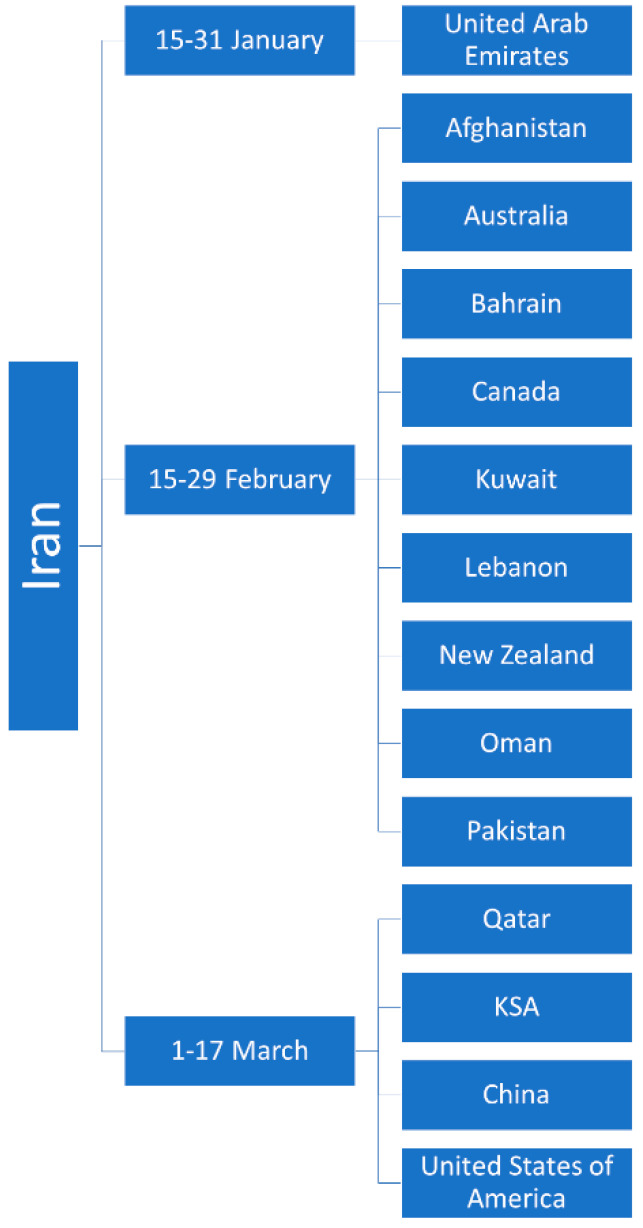
Time periods of imported cases from Iran to other countries.

Figure 3 shows the date in which the countries or cities confirmed their imported cases of COVID-19 from The Islamic Republic of Iran. Of the 70 cases found, 60 cases had reported the dates of entry into each country, and 10 did not report this data. The first cases imported were found to be from 15 January 2020 (UAE), with the latest being imported on 14 March 2020 (Saudi Arabia and China).

### 3.2. Case Characteristics

According to countries with detailed information regarding the imported cases from Iran in Table 1, around 41.4% of the cases were male, 31.4% were female, and 27.1% were either not reported or identified. The ages of reported cases ranged from 21 years up to 70 years. The mean age was not calculatable due to underreporting of demographic data in general. Only 50.0% of the studies reported data about the patient’s age (at least an age range for all their imported cases).

From the articles, only seven articles reported the patient’s symptomology. Overall, only 38.6% of the reported cases were symptomatic, with varied reported symptoms including fever, cough, nasopharyngeal irritation, dyspnea, fatigue/myalgia, headache and diarrhea. On the other hand, 7.1% of patients were asymptomatic. It is important to also note that from the 70 patients reported in the studies, 54.3% did not have any data indicating whether they were symptomatic or asymptomatic.

Worldwide, most countries that detected cases imported from Iran at the airport, by PCR testing alone (24.3%), or both PCR testing and symptomatic screening (20.0%). It is worth noting that for five countries (55.7%, the method of detection was not reported. Some countries (21.4% of cases) reported that detected cases were immediately transferred to quarantine. These countries include Afghanistan, Lebanon, Qatar, Saudi Arabia, New Zealand, and Pakistan. However, not all countries sent the imported cases to quarantine, some were kept in home isolation, such as in cases detected in Canada. Unavoidably, those cases that were not immediately quarantined and kept under home isolation, came in contact with their family and/or friends.

## 4. Discussion

Almost all the recorded first COVID-19 cases in the Eastern Mediterranean Region are imported, and they are mostly from Iran [15]. This highlights the role of travel in spreading infectious diseases between countries and signifies its importance as a potent force in the spread of infections worldwide.

This is the first review to identify which countries had imported cases from Iran, which had become an epicenter for COVID-19, and the main source of spread of the disease to the Middle East [18]. The literature review revealed three main points regarding the imported cases from Iran to the Middle East. Firstly, the imported cases had travelled to Iran for a religious pilgrimage [19,20]. Secondly, tourism between Iran and the UAE also contributed to the spread of the infection, with many Irani tourists visiting the country and carrying the infection with them [21]. Finally, students returning from Iran further expedited the increase in the number of infections within the country [18].

This review shows the number of cases imported, their country of origin, the date of entry and diagnosis, and the symptoms of those who were symptomatic. Moreover, it identifies the method of detection and whether the patients were required to be quarantined or isolated at home. It also identifies the gender and age of the cases. We found that most of the countries carried out airport screenings to detect cases through thermal scans, which largely relies on the infected person being symptomatic in order to test positive for the virus. Nevertheless, this strategy was not effective as shown previously where 80% of the cases are asymptomatic, and there was limited information available about the clinical course of SARS-Cov-2 infection [22]. Furthermore, many countries had different quarantine requirements and testing procedures. Moreover, not all imported cases were detected at the airport, but rather at hospitals. This means that from the date of their arrival until the date of their diagnosis with COVID-19, they have been contributing to the spread of the virus unknowingly. For example, as shown in the table, in Afghanistan, Australia, New Zealand, Pakistan, and Bahrain, the cases were identified at a later time from their arrival. This emphasized the importance of early detection, quarantine, and home isolation procedures at arrival.

Considering the current situation, and for the future lifting of travel restrictions in Qatar, we recommend some measures to be taken for safe travel during this pandemic. Primarily, any city with a rapid spread of the virus should implement an immediate lockdown to avoid any new entries into the country and exit of infected cases. Secondly, travelers should present proof of negative PCR test results before traveling. Third, PCR-testing should be done prior to arrival in the destination country as a confirmation of good health status, and suspected cases should be placed in quarantine immediately. Lastly, promoting general health awareness about the virus, its transmission routes, and manifestation is crucial to raise public awareness about the situation and how to keep themselves safe. Moreover, encouraging people to follow all precautionary measures is imperative to give everyone a sense of responsibility to their community.

Additionally, the Center for Disease Control (CDC) recommends that those returning home from international flights self-quarantine in their homes for at least 14 days [23]. During their quarantine at home, they should monitor their temperature once or twice daily, avoiding contact with others whether it be at home, public transport, or work/school, and consistently practice social distancing.

The main limitation of our study was underreporting of the number of infected cases in the literature results. Many articles were ineligible to be included as they did not quantify the number of confirmed cases or describe their follow-up or state the reasons for travel. Furthermore, there was a drastic gap in the data we collected between the articles we found. Some articles did not provide any information on some of the parameters we were interested in, including comorbidities, age and gender of the cases, the method of detection and the cases’ status after detection, and whether they were quarantined or home isolated. This was mainly because some countries were not reporting their data, hence, we examined gray literature including WHO reports and newspaper articles for further clarification on imported cases. Information on cases were insufficiently communicated by countries to the WHO and the public. Communication failures may lead to delayed outbreak control, diminished public trust and compliance, and unnecessarily prolonged economic, and social disturbances [24].

In conclusion, many countries, especially Arab states and the Middle East, had imported cases from Iran between the 21 February and the 14 March. Our review found that most of the imported cases did not report any symptoms and that PCR testing was the most common method of detection. Additionally, we found that more than half of the imported cases were not quarantined or sent to home isolation after they entered their respective countries, which could have led to these individuals being in contact with their family or friends, resulting in secondary infections. Moreover, many articles had limited data on the characteristics of imported cases. Our main recommendation for countries accepting international travelers is to consign them to quarantine, or at least monitor their home isolation closely to prevent secondary infections, as travelers can often be asymptomatic carriers of COVID-19. Furthermore, updating the public with the current situation of this global pandemic and the measures taken by those countries to contain the spread of the virus is essential. People need to be involved in the fight against this pandemic. They need to be equipped with accurate and transparent information to be able to protect themselves and their families from this evolving infection.

## Figures and Tables

**Table 1:** Summary of cases imported from Iran.

	Reference (Name and Year)	Country	Number of Cases	Country Origin of Imported Cases?	Date of Entry	Date of Diagnosis	Age	Gender	Symptoms (%)	Method of Detection	Quarantined after Detection?
	Mousavi, 2020 [6]	Afghanistan	1	Afghanistan	15-Feb	25-Feb	35 years old	1 Male.	Fever (100%). Headache (100%). Cough (100%). Dyspnea (100%).	RT-PCR	Quarantine
	Riga & Zillman, 2020 [7]	Australia	1	Not reported	24-Feb	28-Feb	63 years old	1 Female.	Symptomatic (100%)	RT-PCR	Not reported
	WHO Event Site for IHR National Focal Event Updates, 2020 [8]	Bahrain	26	22 Bahrain, 4 Saudi Arabia	1st case: 21 Feb	24–26 Feb	Not reported	9 Males. 13 Females. 4 not reported	Not reported	Not reported	Not reported
	“Tracking Every Case of COVID-19 in Canada”, 2020 [9]	Canada	3	Not reported	20–27 Feb	Not reported	Not reported	Not reported	Not reported	Not reported	Home isolation
	Liu, 2020 [10]	China	4	Not reported	Not reported	5–22 Mar	Median 29 years old.	Not reported	Fever (50%), Cough (41.4%), Nasopharyngeal irritation (19.0%), Dyspnea (3.4%), Fatigue/Myalgia (10.3%), Headache (10.3%), Diarrhea (6.9%).	Symptomatic screening. RT-PCR.	Not reported
	“Kuwait report cases of coronavirus”, 2020 & “Kuwait confirm first cases”, 2020 [11]	Kuwait	5	3 Kuwait, 1 Saudi Arabia, 1 not reported	23-Feb	23–24 Feb	21–61 years old	3 Males. 2 Females	Asymptomatic (60%). Fever (40%). Runny nose (20%). Malaise (20%).	Symptomatic screening. RT-PCR.	Not reported
	WHO Event Site for IHR National Focal Event Updates, 2020 & “second coronavirus case recorded in Lebanon”, 2020 [8,12]	Lebanon	3	1 Lebanon, 2 not reported.	20-Feb	21–26 Feb	41 years old, 2 not reported	1 Male. 2 Females	Sore throat (33%). Sneezing (33%). Symptomatic but symptom not reported (66%)	Symptomatic screening. RT-PCR.	Quarantine
	Single case of COVID-19 confirmed in New Zealand [13]	New Zealand	1	Not reported	26-Feb	28-Feb	60 years old	1 Male.	Not reported	RT-PCR	Quarantine
	Coronavirus Disease 2019 (COVID-19) Weekly Situation Report, Issue 1, 2020 [3]	Oman	3	3 Oman	Not reported	Not reported	Not reported	Not reported	Not reported	Not reported	Not reported
	WHO Event Site for IHR National Focal Event Updates, 2020 [8]	Pakistan	2	Not reported	20–23 Feb	26-Feb	22–63 years old	2 Males.	Fever (100%). Headache (100%). Myalgia (100%). Post-nasal drip (50%). Cough (50%).	Not reported	Quarantine
	MOPH, 2020 [14]	Qatar	3	1 Qatar, 2 not reported.	29 Feb–1 Mar	29 Feb–1 Mar	Not reported	1 Male. 2 Unidentified.	Not reported	RT-PCR	Quarantine
	Alah et al, 2020 [15]	Saudi Arabia	5	2 Saudi Arabia, 3 not reported	22 Feb–5 Mar	Not reported	52 years old (1 person)	3 Males. 1 Female. 1 Unidentified.	Not reported	Not reported	Quarantine
	Tayoun et al, 2020 [16]	United Arab Emirates	11	Not reported	15–31 Jan	Not reported	3–70 years old	8 Males. 3 Females	Symptomatic (82%). Asymptomatic (18%).	RT-PCR	Not reported
	Myers et al, 2020 [17]	USA (california)	2	United States	6–17 March	15 April	Not reported	Not reported	Not reported	RT-PCR	Not reported
Totals:	14 references	14 countries	70 cases	1.43% Afghannistan, 2.86% American, 31.42% Bahraini, 1.43% Lebanese, 4.29% Kuwaiti, 4.29% Omani, 1.43% Qatari, 10.00% Saudi Arabian and 42.86% Not reported.	N/A	N/A	N/A	Out of the entire cohort (70 cases) 41.4% males, 31.4% females and 27.1% unidentified cases or not reported	38.6% symptomatic, 7.1% asymptomatic, 54.3% not reported	24.3% PCR alone, 20.0% symptomatic screening & PCR, 55.7% not reported.	21.4% Quarantine, 4.3% home isolation, 74.3% not reported

## Data Availability

Not applicable.
